# Alkaloids as Emerging Neuroprotective Agents for Neurodegenerative Disorders: Insights into Tryptanthrin and Its Derivatives

**DOI:** 10.3390/ijms27167436

**Published:** 2026-08-20

**Authors:** Amjad Khan, Hanif Khan, IL-Ho Park, Badr Abdullah Aldahmash, Muhammad Sohail Khan, Gabsik Yang, Ki Sung Kang

**Affiliations:** 1 College of Korean Medicine, Gachon University, 1342 Seongnamdaero, Seongnam 13120, Republic of Korea; 2 Department of Neurosurgery, Penn State Hershey Medical Center, Hershey, PA 17033, USA; 3 Department of Radiation Oncology, Penn State Cancer Institute, Hershey Medical Center, Hershey, PA 17033, USA; 4 College of Pharmacy, Sahmyook University, Seoul 01795, Republic of Korea; 5 Department of Zoology, College of Science, King Saud University, P.O. Box 2455, Riyadh 11451, Saudi Arabia

**Keywords:** neurodegenerative diseases, alkaloids, typtanthrin, tryptanthrin derivatives, Alzheimer’s disease, Parkinson’s disease, spinal cord injury, inflammation, oxidative stress

## Abstract

Neurodegenerative conditions are incurable, progressive disorders characterized by the slow and irreversible loss of neurons. This neuronal loss can lead to several neuropsychiatric disorders and long-term complications. Despite significant advances in understanding the mechanisms of neurodegenerative disease, currently, there is no cure for neurodegenerative diseases, highlighting the urgent need for novel neuroprotective strategies. Alkaloids are an important class of bioactive substances that exhibit neuroprotection against several neurodegenerative diseases. Tryptanthrin, an indoloquinazoline alkaloid, shows strong anti-inflammatory, antioxidant, and neuroprotective properties. In this review, we described the pathophysiology of neurodegenerative diseases and summarized several alkaloids’ neuroprotective properties. Furthermore, we showed protective benefits of tryptanthrin and its derivatives against neurodegenerative illnesses, focusing on their modulation of oxidative stress, neuroinflammation, neuronal death, and related signaling pathways in cellular and animal models of neurodegenerative diseases. However, various challenges, such as clinical evidence, pharmacokinetic studies, and long-term treatment effects, are not well documented. Future research on tryptanthrin and its derivatives should focus on the optimization of drug delivery methodologies and clinical studies to establish its potential as a therapeutic candidate for neurodegenerative diseases.

## 1. Introduction

The term neurodegeneration originates from the words “neuron,” referring to nerve cells, and “degeneration,” which denotes progressive deterioration or loss, collectively describing the loss of neuronal structure and function [1]. Neurodegenerative disorders (NDs) have become a major global health burden because effective treatments remain unavailable. These diseases are among the leading causes of disability and premature death worldwide, and their incidence is expected to increase substantially over the coming decades [2,3]. NDs are commonly associated with the progressive deterioration of synapses and neurons, usually occurring later in life. Although the precise causes of NDs remain unknown, proteolytic stress, neuroinflammation, oxidative stress, mitochondrial dysfunction, and aging contribute to neuronal cell death and the development of conditions such as Alzheimer’s disease (AD), Parkinson’s disease (PD), Huntington’s disease (HD), and Amyotrophic Lateral Sclerosis (ALS). Among these disorders, AD and PD are the most prevalent and account for the highest number of neurodegeneration-related deaths worldwide [4,5,6,7]. According to the 2021 Global Burden of Disease (GBD) study, approximately 21.8 million (19.1–24.8) individuals worldwide were living with dementia in 1990, compared to 56.9 million (49.4–65.0) in 2021. Similarly, the GBD study reported a continuous increase in the number of individuals living with PD. In 1990, around 3.1 million (2.7–3.6) people were estimated to have PD, whereas by 2021, the number had increased to roughly 11.8 million (10.4–13.4) worldwide [8].

**Figure 1:**
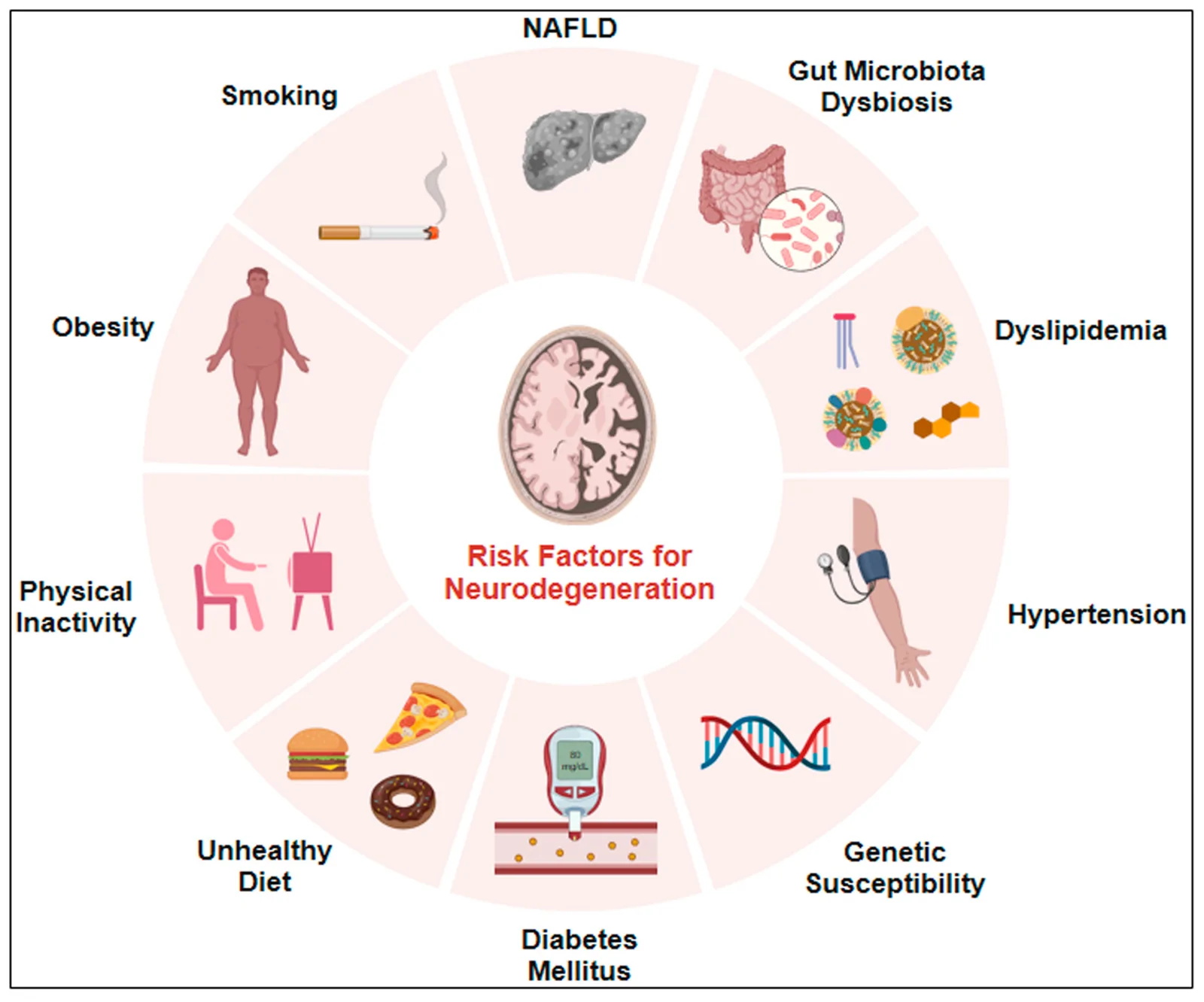
Risk factors involved in neurodegeneration. Schematic representation of the major risk factors and causes contributing to neurodegeneration. Multiple factors, including aging, genetics, diet, diabetes, lifestyle-related influences, and several others, initiate and exacerbate pathological processes in the central nervous system.

Several factors contribute to neurodegenerative conditions. The accumulation of toxic proteins in the brain activates multiple harmful pathways, including oxidative stress (OS), which plays an important role in the pathogenesis of NDs. Because the brain has a high oxygen demand and relatively weak antioxidant defense systems, it is particularly susceptible to OS. Due to an imbalance between the generation of reactive oxygen species (ROS) and the body’s ability to remove them, the brain becomes vulnerable to oxidative damage [9,10,11]. Elevated ROS levels can cause cellular dysfunction and neurodegeneration. Furthermore, ROS-induced cellular damage in the central nervous system (CNS) can trigger neuroinflammation, a key pathological feature of NDs [12]. Local immune cells, such as microglia and astrocytes, become activated under these pathological conditions and produce pro-inflammatory mediators and cytokines, including interleukin-1β (IL-1β), interleukin-6 (IL-6), and tumor necrosis factor-α (TNF-α) [11,13]. The release of these toxic factors can accelerate chronic inflammation and neuronal dysfunction. This inflammatory state in the CNS impairs synaptic function, promotes neuronal loss, and contributes to neurodegeneration. Consequently, OS and neuroinflammation interact to create a self-propagating toxic environment in the brain that significantly contributes to the initiation and progression of NDs [14,15]. The major underlying causes of NDs are illustrated in Figure 1.

Several pharmacotherapeutic agents for neurodegeneration are available on the market; however, none effectively cure the disease or halt its progression. Consequently, the discovery of new and effective medicines is necessary to protect human health against these conditions. Natural compounds exhibit a broad spectrum of pharmacological properties and have been extensively evaluated in experimental models of NDs, where they have demonstrated significant neuroprotective and therapeutic effects [16,17]. Among them, alkaloids represent a diverse group of natural compounds with various pharmacological activities and have been extensively studied for the treatment and prevention of various NDs. Alkaloids are distinctive, naturally occurring, specialized metabolites characterized by the presence of nitrogen in their chemical structures. They exhibit diverse chemical structures and functional properties [18]. Alkaloids are found in marine organisms, insects, and plants [19,20]. Their diversity and widespread distribution have increased their use and attracted attention in modern medicine for the treatment of chronic diseases such as diabetes, cancers, and NDs. Tryptanthrin is an indoloquinazoline alkaloid isolated from several natural sources. It has been reported to modulate important signaling pathways, including oxidative and inflammatory pathways. In addition, it exhibits neuroprotective properties against NDs [21,22,23,24,25,26].

## 2. Literature Search Strategy

We extensively reviewed the articles showing the beneficial effects of Alkaloids, tryptanthrin, and its derivatives in different diseases and several models of neurodegenerative diseases. Relevant peer-reviewed articles published in English were identified through comprehensive searches of major scientific databases, including PubMed, Web of Science, Scopus, and Google Scholar. Literature published up to 2026 was identified using different keywords, such as alkaloids, tryptanthrin, natural products, neuroprotection, and tryptanthrin and its derivatives. Neurodegenerative diseases, Alzheimer’s disease, Parkinson’s disease, oxidative stress, and neuroinflammation. Original research articles, preclinical studies, mechanistic investigations, and relevant review articles were considered to provide a comprehensive overview of the field. Priority was given to studies investigating the pharmacological activities, molecular mechanisms, signaling pathways, and therapeutic potential of tryptanthrin and related alkaloids in cellular and animal models of several diseases and neurodegeneration. The available literature was critically analyzed and synthesized to highlight current knowledge, emerging therapeutic perspectives, and existing research gaps.

## 3. Overview and Classification of Alkaloids

The term “alkaloid” was derived from the Arabic name al-qali, coined by the German scientist Carl F.W. Meissner in 1819. Alkaloids are a diverse group of naturally occurring chemical compounds that generally contain at least one nitrogen atom, which is considered a characteristic chemical feature of this class of compounds [27,28]. Although alkaloids are generally basic compounds, certain alkaloids may be neutral or weakly acidic. Some alkaloids may contain sulfur and rarely phosphorus, bromine, or chlorine. Alkaloids exhibit solubility in water in acidic conditions, but they dissolve in lipids in basic and neutral environments [29]. Alkaloids are predominantly found in plants; however, they are also present in animals, insects, microorganisms, and marine organisms. Alkaloids are pharmacologically active compounds that exert protective effects against various diseases [30]. Alkaloids can be classified in various ways based on their chemical and biosynthetic diversity. The most common classification is based on their biosynthetic origin, nitrogen atom location, and core chemistry or heterocyclic ring structure. These are described in detail below.

### 3.1. Classification Based on the Position of the Nitrogen Atom

Alkaloids are classified as heterocyclic or nonheterocyclic according to where the nitrogen atom is in the chemical structure. Heterocyclic alkaloids have at least one nitrogen atom as a component of the heterocyclic ring. Nonheterocyclic alkaloids, also known as protoalkaloids, have a nitrogen atom outside the ring structure, typically within an aliphatic side chain [19].

### 3.2. Classification Based on Chemical Ring Structure

Alkaloids can also be classed based on their distinctive core ring or heterocyclic structure. The main structural classes include piperidine, isoquinoline, tropane, quinoline, purine, pyrrolizidine, indole, imidazole alkaloids, and Indoloquinazoline alkaloids.

#### 3.2.1. Piperidine Alkaloids

Piperidine alkaloids have a six-membered saturated heterocyclic ring consisting of five carbon atoms and one nitrogen atom, known as the C5N ring system. Piperidine and its derivatives are found in many plant species and form a significant structural group of alkaloids. Typical examples include prosopinine, sedamine, adaline, and piperine-related substances [31].

#### 3.2.2. Isoquinoline Alkaloids

Isoquinoline alkaloids contain an isoquinoline or structurally related tetrahydroisoquinoline nucleus. Many plant families, including the Annonaceae, Berberidaceae, Fabaceae, Lauraceae, Menispermaceae, and Papaveraceae, contain Isoquinoline alkaloids. Notable members of this class include morphine, codeine, papaverine, and berberine [32].

#### 3.2.3. Tropane Alkaloids

Tropane alkaloids have a bicyclic tropane nucleus consisting of a nitrogen-containing bridging ring structure. These are significant secondary metabolites that are mostly present in members of the Erythroxylaceae and Solanaceae families. Cocaine, scopolamine, hyoscyamine, anisodamine, and calystegines are examples of tropane alkaloids [33].

#### 3.2.4. Quinoline Alkaloids

Quinoline alkaloids include a quinoline or similar heterocyclic nucleus. Quinine, which is derived mostly from the bark of cinchona species, is one of the most well-known members of this class. Camptothecin, derived from Camptotheca acuminata, is another pharmacologically significant molecule of this group [25].

#### 3.2.5. Purine Alkaloids

Purine alkaloids, which are based on a purine-derived ring structure, are found in many plants. Caffeine, theobromine, and theophylline are some of the most prevalent examples. These chemicals are found in plants from the genera Coffea, Camellia, Theobroma, Cola, Paullinia, and some Citrus species [34].

#### 3.2.6. Pyrrolizidine Alkaloids

Pyrrolizidine alkaloids consist of a pyrrolizidine nucleus made up of two fused five-membered rings that share a nitrogen atom. They are found in several plant families, including the Asteraceae, Boraginaceae, and Apocynaceae, as well as some genera of the Orchidaceae and Fabaceae. Representative examples include senecionine, retrorsine, lycopsamine, monocrotaline, heliotrine, and intermedine [35].

#### 3.2.7. Indole Alkaloids

Indole alkaloids are one of the most abundant types of naturally occurring alkaloids, and are characterized by the presence of an indole ring in their chemical structure. The most commonly found indole alkaloids include indolamines, erchinines, kopsihainins, mitragynine, melokhanines, and brucine [36].

#### 3.2.8. Imidazole Alkaloids

Imidazole alkaloids have an imidazole ring as their major structural characteristic. They have been found in both terrestrial and marine animals, such as plants, sponges, mussels, and other marine invertebrates. Pilocarpine is a popular plant-based imidazole alkaloid [37].

#### 3.2.9. Indoloquinazoline Alkaloids

Indoloquinazoline alkaloids are distinguished by a combined heterocyclic system that includes both indole and quinazoline ring members. Tryptanthrin, scientifically described as indolo[2,1-b]quinazoline-6,12-dione, is a natural indoloquinazoline alkaloid isolated from a variety of therapeutic plants and microbes [38].

## 4. Pharmacological Properties of Alkaloids

Alkaloids are a structurally varied group that have a wide range of pharmacological effects via interactions with receptors, enzymes, ion channels, and intracellular signaling pathways. Alkaloids, due to their extraordinary chemical diversity, have been widely used to treat neurological, metabolic, cardiovascular, neoplastic, infectious, and inflammatory disorders. Some alkaloids’ activities are described below.

### 4.1. Analgesic Activities

Some alkaloids have significant analgesic and neuromodulating effects. Papaver somniferum contains morphine and codeine, which are traditional opioid analgesics that predominantly activate opioid receptors. Morphine is still one of the most effective medications for severe pain treatment, but codeine is routinely used for moderate to mild pain and cough reduction. Despite its therapeutic efficacy, chronic use can cause breathing problems, tolerance, dependency, and opiate addiction. High doses cause death if not maintained properly due to respiratory failure [39,40]. Papaverine, a benzylisoquinoline alkaloid derived from Papaver somniferum, is distinct from morphine; it lacks opioid receptor function. It is isolated from Papaver somniferum, a plant belonging to the family Papaveraceae. It is well known for its smooth muscle relaxant and vasodilatory properties [41].

### 4.2. Anti-Inflammatory and Antioxidant Activities

Berberine is extracted from the stem-bark and roots of several berberis plants that are members of the Berberidaceae family. Berberine has beneficial effects in metabolic disorders, cardiovascular illnesses, diabetes, and inflammatory conditions [42]. A study showed that berberine increases AMP-activated protein kinase (AMPK), inhibits TLR4/MD-2 signaling, and lowers inflammatory cytokine production. Berberine also possesses antihypertensive, anti-inflammatory, antioxidant, and hepatoprotective properties, making it a promising therapeutic agent for the management of various metabolic and inflammatory disorders [43,44,45]. Capsaicin is an active alkaloid-like compound isolated from the fruits of Capsicum species, such as Capsicum annuum and Capsicum frutescens, belonging to the family Solanaceae. It acts on sensory neurons by binding to the transient receptor potential vanilloid 1 (TRPV1) receptor, its primary molecular target. Although capsaicin is well known for its analgesic effects, it also has anti-inflammatory and antioxidant capabilities via regulation of sensory neuropeptides and inflammatory mediators [46,47].

### 4.3. Cardiovascular and Autonomic Activities

Several alkaloids have therapeutic significance due to their effects on the cardiovascular and autonomic nerve systems.

Quinine, obtained from the bark of Cinchona species, has historically served as an effective antimalarial agent [48]. Its stereoisomer quinidine is commonly used as a class IA antiarrhythmic medication. Quinidine inhibits voltage-gated cardiac sodium channels and delays repolarization by blocking potassium ions, prolonging cardiac action potentials [49,50]. Reserpine, isolated from Rauwolfia species, permanently inhibits vesicular monoamine transporter-2 (VMAT2), resulting in catecholamine and serotonin depletion. As a result, it has been prescribed as an antihypertensive and antipsychotic drug, but its therapeutic usage decreased due to adverse effects on the central nervous system [51]. Ergotamine is isolated from the seeds of Claviceps purpurea, belonging to the family Clavicipitaceae [52]. It acts on serotonergic, dopaminergic, and adrenergic receptors and is used for migraine treatment due to its vasoconstrictive properties [53,54].

Yohimbine is an indole alkaloid obtained from the bark of Pausinystalia yohimbe, a plant belonging to the family Rubiaceae. It functions as a selective α2-adrenergic receptor antagonist. Pharmacologically, it is known to increase blood pressure and heart rate due to its sympathomimetic effects [55,56].

### 4.4. Antineoplastic (Anticancer) Activities

Several plant-derived alkaloids possess potent antineoplastic properties. Vinblastine is a vinca alkaloid isolated from the pink periwinkle plant, Catharanthus roseus, belonging to the family Apocynaceae. It is used for breast cancer, Kaposi sarcoma, renal cell carcinoma, and testicular cancer [57]. Vincristine is another vinca alkaloid commonly used as a chemotherapy drug for the treatment of leukemia, lymphoma, myeloma, breast, head, and neck cancer. Vindesine, a semisynthetic derivative of vinblastine, is used to treat some cancers, the most common of which is acute lymphocytic leukemia. Colchicine has been reported to have antitumor effects on gastric carcinoma cell lines, colon cancer cells, and human breast adreno-carcinoma MCF-7 cells [58,59]. Berberine has been shown to exhibit anticancer properties. Berberine consistently regulates miRNA expression in preclinical breast cancer models, helping to inhibit tumor-related cellular pathways. It has also shown anticancer effects in ovarian, bladder, and liver cancers [60]. Coptisine is an isquinoline alkaloid mainly extracted from Coptis chinensis. Coptisine has shown considerable anticancer efficacy in hepatocellular carcinoma [61]. Matrine is an alkaloid extracted from the root of Sophora flavescens. It shows anticancer properties in a variety of cancers. Matrine showed anticancer effects in lung cancer cells, in colorectal cancer, and inhibits the growth of acute myeloid leukemia [62].

## 5. Etiology of Neurodegenerative Diseases

Several complex pathological mechanisms mediate various neurodegenerative diseases and conditions and ultimately lead to neuronal loss, impaired communication between cells, disruption of normal cellular function, and decline in cognitive and motor functions. Some of the neurodegenerative diseases and their etiological factors are discussed below.

### 5.1. Alzheimer’s Disease

Alzheimer’s disease (AD) is the most common neurodegenerative disorder and is characterized by progressive cognitive decline and memory impairment. Its etiology is multifactorial, involving aging, genetic susceptibility, environmental factors, and alterations in cellular homeostasis [63,64]. The major pathological features of AD include the accumulation of extracellular amyloid-beta (Aβ) plaques and the hyperphosphorylation of tau protein. These pathological changes contribute to synaptic dysfunction and neuronal degeneration [65,66]. The accumulation of these toxic proteins induces oxidative stress and mitochondrial dysfunction [67]. Oxidative stress and mitochondrial dysfunction further contribute to AD progression by increasing reactive oxygen species (ROS) production, disrupting cellular energy metabolism, and accelerating neuronal damage. In addition, activation of glial cells occurs in AD, contributing to neuroinflammation and neurodegeneration. Activated glial cells release inflammatory mediators and cytokines, such as TNF-α, interleukin-1β (IL-1β), and COX-2, which further exacerbate neuroinflammation and neuronal damage [68]. Together, these pathogenic processes contribute to synaptic dysfunction and neuronal death, ultimately leading to learning and memory impairment and cognitive dysfunction.

### 5.2. Parkinson’s Disease

Parkinson’s disease (PD) is the second most common neurodegenerative disease characterized by selective degeneration of dopaminergic neurons in the substantia nigra pars compacta (SNpc) and impairment of dopaminergic neurotransmission [69,70]. Damage to these neurons decreases dopamine levels in the nigrostriatal pathway, causing motor symptoms such as bradykinesia, stiffness, resting tremor, and postural instability. Accumulation of alpha-synuclein (α-Syn) within neurons is a major pathologic hallmark of PD. Alpha-synuclein misfolds and aggregates abnormally, leading to the formation and accumulation of Lewy bodies in the brain. These pathological changes are associated with mitochondrial dysfunction [71]. Neurotoxins, such as MPTP (1-methyl-4-phenyl-1, 2, 3, 6-tetrahydropyridine), can impair mitochondrial complex I activity, resulting in reduced ATP production, increased ROS generation, and oxidative damage [72,73]. Neuroinflammation is also an important contributor to the pathogenesis of PD. Alpha-synuclein triggers glial activation, which further releases inflammatory mediators, including TNF-α and IL-1β, which can promote inflammation and neuronal injury [74,75]. Collectively, multiple factors, including alpha-synuclein aggregation, mitochondrial dysfunction, oxidative stress, and neuroinflammation, contribute to the loss of dopaminergic neurons and associated motor symptoms of PD.

### 5.3. Huntington’s Disease (HD)

Huntington’s disease (HD) is a rare inherited neurodegenerative disorder characterized by progressive motor dysfunction, cognitive decline, and behavioral abnormalities [76]. HD is caused by an abnormal expansion of CAG repeats in the huntingtin gene (HTT), resulting in the production of mutant huntingtin protein (mHTT) with an expanded polyglutamine tract at its N-terminus. HTT is a large protein that plays important roles in several cellular functions, including intracellular transport and cell survival. Mutant HTT can form toxic oligomers and intracellular inclusion bodies, which are characteristic pathological features of HD [77]. The accumulation of mHTT contributes to transcriptional dysregulation, mitochondrial dysfunction, oxidative stress, excitotoxicity, and impaired autophagy. Moreover, abnormal glutamatergic signaling and calcium dysregulation can promote excitotoxic neuronal damage. These interconnected mechanisms contribute to the degeneration of medium spiny neurons (MSNs) in the striatum, ultimately leading to progressive motor, cognitive, and behavioral impairments [78,79].

### 5.4. Traumatic Brain Injury (TBI)

Traumatic brain injury (TBI) is a disruption of the normal structure and function of the brain caused by an external mechanical force, such as a bump, blow, or jolt to the head or penetration of brain tissue by an object [80]. Common causes of TBI are falls, blast-related traumatic brain injuries, road traffic accidents and high-impact sports such as boxing, soccer, hockey, and skateboarding [81]. TBI is a major cause of mortality and long-term disability worldwide [82]. The clinical symptoms of brain injury consist of headache, seizures, coma, nausea, and behavioral changes. The prolonged complication is cognitive and memory impairments [83]. TBI involves both primary and secondary injury mechanisms. Primary injury occurs at the time of trauma as a direct consequence of mechanical forces and may cause structural tissue damage, vascular injury, and hemorrhage. Secondary injury develops following the initial insult and can progress from minutes to months, involving a cascade of pathological processes that contribute to neuronal cell death, neuroinflammation, and neurodegeneration [84].

Despite their different etiologies and clinical characteristics, AD, PD, HD, and TBI all share multiple interrelated pathogenic processes, such as oxidative stress, neuroinflammation, defective autophagy, mitochondrial dysfunction, excitotoxicity, and apoptosis [85,86]. Aging, as well as environmental and lifestyle variables, may increase oxidative stress, neuroinflammation, and neuronal apoptosis [87].

## 6. Alkaloids and Neuroprotection

Many naturally occurring alkaloids have shown interesting neuroprotective properties in experimental studies of neurodegenerative conditions. Preclinical investigation shows that alkaloids target a variety of pathogenic mechanisms, including neuroinflammation, oxidative stress, mitochondrial malfunction, protein aggregation, and neuronal death. The following sections outline the neuroprotective effects of certain alkaloids in various neurodegenerative disorders.

### 6.1. Neuroprotective Effects of Alkaloids in Alzheimer’s Disease

Several alkaloids have shown protective properties in curing of AD complications.

Berberine exhibits various neuroprotective effects in mouse models of AD. It reduced Aβ plaque accumulation in the cortex and hippocampus, decreased neuroinflammation, and improved memory function in 5xFAD mice [88]. Evodiamine has been shown to exert protective effects in animal models of neurodegeneration. Evodiamine significantly inhibited Tau and GSK3β hyperphosphorylation, reduced oxidative stress, and improved impaired spatial memory and learning in mice [89]. Similarly, in an AD model, evodiamine suppressed glial activation and decreased the levels of pro-inflammatory cytokines, thereby mitigating neuroinflammation in the brain [90]. Palmatine prevents Aβ-induced paralysis and enhances antioxidant defenses in Aβ-transgenic *Caenorhabditis elegans* [91]. Tetrandrine treatment in 5XFAD mice reduced Aβ plaque deposition in the brain and downregulated inflammation-associated genes, including TNF-α, IL-1β, IL-6, cyclooxygenase-2 (COX-2), inducible nitric oxide synthase (iNOS), and p65. These findings demonstrate its potential role in attenuating neurotoxicity, neuroinflammation, and neurodegeneration [92]. Galanthamine prevents Aβ-induced oxidative neuronal damage in cultured rat cortical neurons [93]. Huperzine demonstrated multiple anti-Alzheimer’s targets by combining cholinergic, neuroprotective, and disease-modifying pathways. In transgenic AD mice, huperzine treatment enhanced spatial learning and memory, and decreased hippocampus amyloid-β plaque load. It increases choline acetyltransferase activity, reduces oxidative stress, and suppresses apoptosis. It also showed significant antioxidative and anti-inflammatory properties by scavenging reactive oxygen species and modulating neuroinflammatory pathways [94].

Nicergoline, an ergoline alkaloid, has been shown to improve memory and cognitive function in 3xTg-AD mice. In addition, it also reduced hippocampal apoptosis, oxidative stress, and neuroinflammation [95].

### 6.2. Neuroprotective Effects of Alkaloids in Parkinson’s Disease

In Parkinson’s disease (PD), several alkaloids showed their protective actions in several models.

Berberine showed strong neuroprotective properties in MPTP-induced Parkinson’s disease mice; it protects against dopaminergic neuronal degeneration in the substantia nigra and enhances motor performance [96].

Rhynchophylline is an indole alkaloid that exhibited neuroprotective effects in MPTP-induced PD mice by restoring spontaneous motor functions. In addition, it inhibited MPP^+^-induced cell death in PC12 cells in vitro [97]. Piperine in 6-OHDA-induced PD rats improved autonomic movement and gastrointestinal dysfunctions. It further prevented the loss of dopaminergic neurons and reduced α-Syn aggregation [98]. Piperine significantly enhanced motor impairments and prevented dopaminergic neuron loss in a rotenone-induced PD animal model. In vitro, piperine increased cell viability and restored mitochondrial function in rotenone-treated SK-N-SH cells and primary neuronal cultures [99]. In another 6-hydroxydopamine (6-OHDA)-induced PD rat model, berberine improved motor functions and prevented the decline of the number of tyrosine-hydroxylase (TH)-positive neurons [100].

### 6.3. Neuroprotective Effects of Alkaloids in Huntington Disease

In some animal and cellular models of Huntington’s disease (HD), alkaloids also showed their protective properties. In HD, mice treated with morphine showed improved analgesic effects. Following morphine injection, astrocytes and microglial cells in the spinal cord of premanifest HD mice were less activated. Additionally, HD mice exhibited lower levels of the inflammatory cytokines TNF-α and IL-1β than wild-type (WT) animals [101]. Berberine reduced the accumulation of mutant huntingtin in cultured cells. Berberine treatment in transgenic N171-82Q-HD mice alleviated motor dysfunction and prolonged survival. It also increases the degradation of mutant huntingtin by enhancing autophagic function [102].

### 6.4. Neuroprotective Effects of Alkaloids in Traumatic Brain Injury

Huperzine A improved cognitive impairment in mice with traumatic brain injury (TBI), reduced brain edema and OS in the cortex, promoted Nrf2 nuclear translocation, and increased synaptic protein expression [103]. Berberine has shown neuroprotective effects in both in vitro and in vivo TBI models. Berberine was delivered into mice that had undergone controlled cortical impact damage. Berberine effectively reduced functional impairments and brain damage caused by TBI. Berberine also decreased neuronal death, apoptosis, blood–brain barrier (BBB) permeability, and cerebral edema. Berberine inhibited TLR4, MyD88, and NF-κB activation in mixed glial cultures [104]. Tetrandrine showed neuroprotective properties in the TBI model. Tetrandrine enhanced neurological function, reduced brain edema and BBB disruption, inhibited neuroinflammation by downregulating TNF-α, NF-κB, and TRAF1 expression, and inhibited neuronal apoptosis by decreasing caspase-3 and caspase-12 in both in vitro and mouse TBI models [105].

Taken together, the above evidence indicates that alkaloids confer neuroprotection by targeting multiple neurodegenerative diseases (NDs) and pathways. However, several limitations should be considered when interpreting these preclinical findings. Current in vitro and animal model studies may not accurately reflect the complexities of NDs in humans. Moreover, variations in experimental design, therapeutic dosing protocols, and disease models complicate direct comparisons across studies. In addition, concerns regarding absorption, blood–brain barrier permeability, and long-term safety remain insufficiently addressed. Therefore, despite promising preclinical findings, further research is required to confirm the therapeutic potential of alkaloids in clinical settings.

## 7. Tryptanthrin and Its Derivatives/Analogues: Biological Activities, Sources, and Pharmacological Profile

Tryptanthrin, an alkaloid belonging to the indoloquinazoline class (indolo [2,1-b]quinazoline-6,12-dione, 6), contains an indole ring fused to a quinazoline ring with carbonyl groups at the 6- and 12-positions. Several naturally occurring and chemically synthesized tryptanthrin derivatives have been characterized by diverse substitutions at the 6-position of the indolo [2,1-b]quinazoline core structure [106,107,108,109]. Table 1 summarizes the chemical structures of some tryptanthrin derivatives described in previous studies.

**Figure 2:**
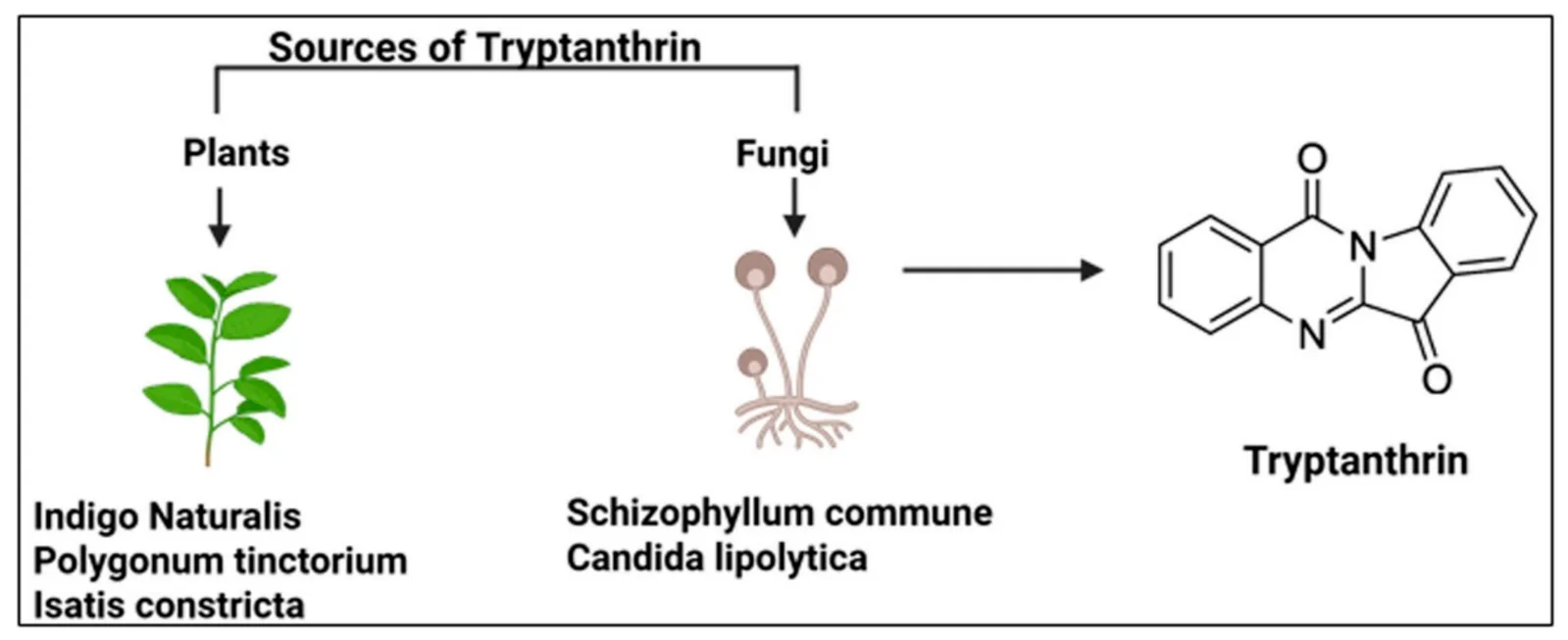
Natural sources and chemical structure of tryptanthrin. Tryptanthrin is an indoloquinazoline alkaloid isolated from various natural sources, including medicinal plants and microorganisms such as fungi, along with its characteristic chemical structure, indoloquinazoline class (indolo [2,1-b]quinazoline-6,12-dione, 6).

**Figure 3:**
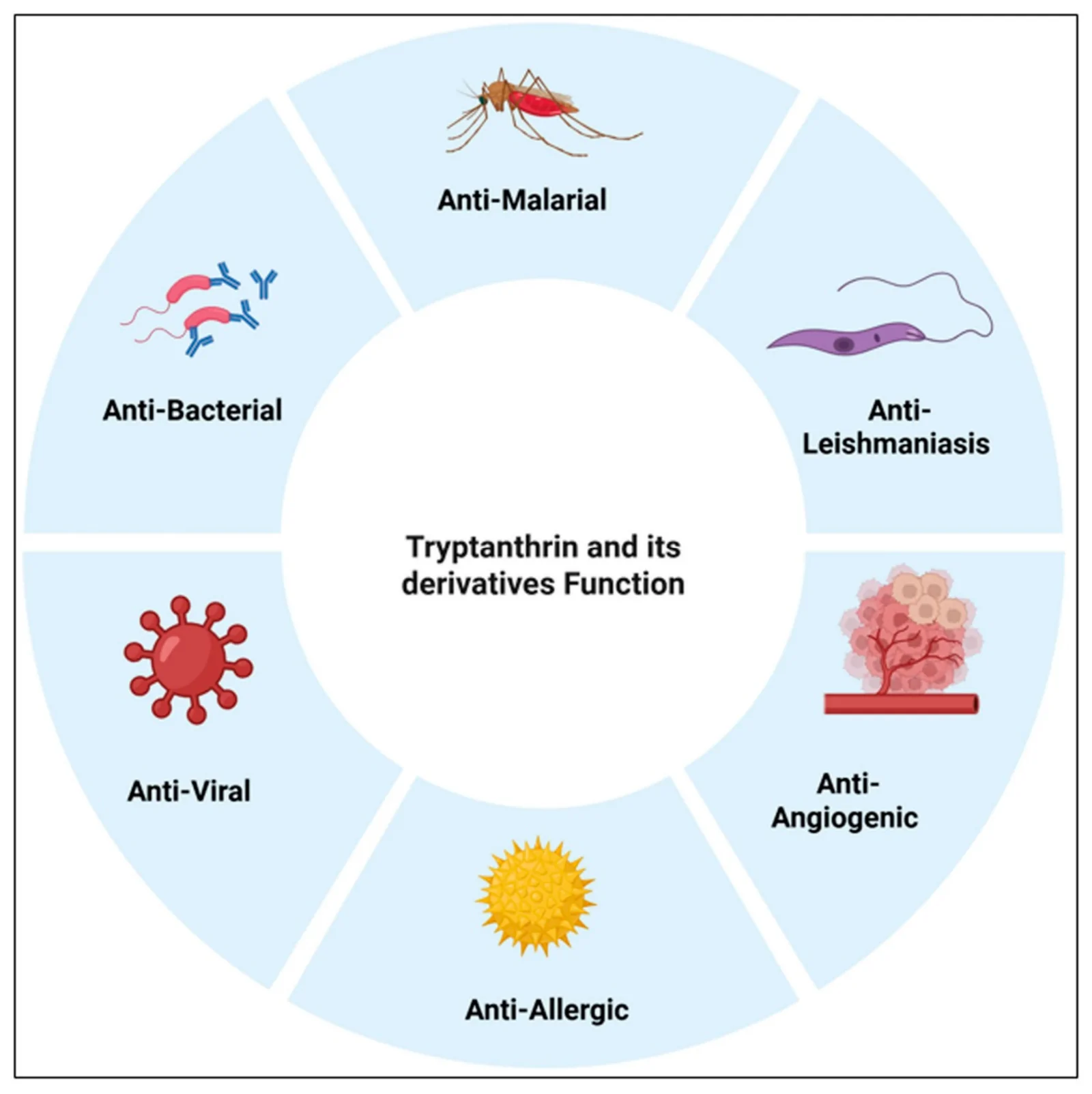
Pharmacological activities of tryptanthrin and its derivatives. Overview of the different pharmacological activities of tryptanthrin and its derivatives. Tryptanthrin exhibits a broad spectrum of biological functions, including antibacterial, antiviral, antimalarial, anti-leishmanial, anti-allergic, and anti-angiogenic activities. These multifunctional properties highlight its therapeutic potential in various infectious disorders.

Tryptanthrin is found in diverse natural sources, including yeast, fungi, and several plant species, as shown in Figure 2 [116,119]. It has been isolated from indigo-bearing traditional Chinese herbal medicines, such as Indigo Naturalis, Isatidis Radix, and Isatidis Folium [120]. Recent studies have shown that it exhibits several therapeutic activities, including anti-phytopathogenic bacterial [121], antibacterial [122], anti-viral [123], anti-phytopathogenic fungal [124], anti-allergic [125], anti-angiogenic in human vascular endothelial cells [126], anti-leishmanial [21], and antimalarial [127]. It also reduced erosive lesions and inflammation in a mouse model of colitis [128]. A broad spectrum of biological activities has been reported for tryptanthrin analogues. A series of novel tryptanthrin derivatives was synthesized and evaluated for their inhibitory effects against human cancer cell lines, including lung (A549), chronic myeloid leukemia (K562), prostate (PC3), and liver (HepG2) [129]. Another series of tryptanthrin derivatives showed anti-tumor activity against human hepatocellular carcinoma cells [130]. Tryptanthrin derivatives containing benzenesulfonamide substituents showed anti-inflammatory and antioxidant activities [131,132]. The different protective activities of tryptanthrin and its derivatives are shown in Figure 3.

## 8. Neuroprotective Effects of Tryptanthrin and Its Derivatives in Neurodegenerative Diseases

Tryptanthrin has shown neuroprotective properties against several NDs, some of which are listed below.

### 8.1. Tryptanthrin/Derivatives in Alzheimer’s Disease

Tryptanthrin derivatives containing benzenesulfonamide substituents have shown multifunctional potential for the treatment of Alzheimer’s disease (AD). Guoxing Wang et al. reported that a tryptanthrin derivative, designated **compound** **4h**, exhibited neuroprotective effects against H_2_O_2_-induced PC12 cell injury. Transmission electron microscopy (TEM) analysis and thioflavin T (ThT) fluorescence analyses showed that treatment with **4h** considerably inhibited the self-aggregation of Aβ_1–42_ peptides, with an inhibition rate of 63.16% ± 2.33%. Molecular docking results demonstrated that **4h** efficiently bound to Aβ, stabilized the α-helical structure, and prevented the toxic conformation of Aβ_1–42_. Furthermore, in a scopolamine-induced AD mouse model, administration of **4h** for seven consecutive days improved memory and cognitive function in a dose-dependent manner [133]. Tau protein aggregation in the brain is a major hallmark of AD, and this study showed that **4h** inhibited tau protein aggregation. Another study reported that tryptanthrin derivatives inhibited both seeded and unseeded tau protein aggregation, suggesting that these compounds may play an important role in preventing toxic tau aggregation in AD [134].

### 8.2. Neuroprotective Properties of Tryptanthrin-6-Oxime in a Rat Model of Transient Focal Cerebral Ischemia

In a rat model of transient focal cerebral ischemia, tryptanthrin 6-oxime protected the brain against ischemic injury. In a dose-dependent manner, treatment with 5 and 10 mg/kg significantly improved neurological function, reduced brain infarct volume, and decreased brain edema after stroke [135].

### 8.3. Tryptanthrin and Neuroinflammation

Neuroinflammation plays an important role in NDs. During neuroinflammation, glial cells become activated and release proinflammatory cytokines that contribute to neuronal damage. Tryptanthrin and its derivatives have been reported to reduce inflammation and the production of pro-inflammatory mediators. IL-1β, TNF-α, and nitric oxide (NO) are key inflammatory mediators involved in neuroinflammation. In LPS-stimulated BV2 cells, tryptanthrin derivatives significantly inhibited the production of NO, IL-1β, TNF-α, and COX-2 in a concentration-dependent manner [136]. Tryptanthrin, isolated from Polygonum tinctorium Lour, demonstrated neuroprotective effects in LPS-stimulated BV2 microglial cells. In this study, several inflammation-related markers, including iNOS, COX-2, toll-like receptor 4 (TLR4), and nuclear factor kappa B (NF-κB p65), were examined. LPS treatment significantly increased the expression of iNOS, COX-2, TLR4, and NF-κB in BV2 microglial cells, whereas tryptanthrin treatment markedly reduced the expression levels of these inflammatory proteins [137]. Another study found that LPS induced a pro-inflammatory M1-like phenotype in BV2 cells. In a dose-dependent manner, tryptanthrin treatment prevented the LPS-induced morphological changes in BV2 cells. Furthermore, tryptanthrin also inhibited LPS-induced inflammation in the BV2 cell line [138].

### 8.4. Tryptanthrin’s Role in Spinal Cord Injury

Tryptanthrin also exhibited neuroprotective effects against spinal cord injury. It targets the cGAS/STING/NF-κB pathway to induce microglial polarization toward the M2 phenotype and promote functional recovery in a mouse model of spinal cord injury. In this study, tryptanthrin (3 μg/mL; 30 mg/kg/day for 14 days) was administered intragastrically to mice with spinal cord injury. The authors found that tryptanthrin facilitated tissue repair and functional recovery after spinal cord injury. Footprint analysis showed that stride length was significantly improved in tryptanthrin-treated mice. Tryptanthrin also reduced neuronal loss after spinal cord injury by preserving ventral motor neurons (VMNs), which are essential for locomotor recovery. Nissl and NeuN staining confirmed that tryptanthrin markedly reduced VMN loss, inhibited neuronal apoptosis, and promoted neuronal survival [138].

### 8.5. Tryptanthrin/Derivatives and Oxidative Stress

Tryptanthrin and its derivatives have also shown protective effects against oxidative stress in cellular and animal models, as described below.

In BV2 microglial cells, LPS induces OS and ROS production. Tryptanthrin activated the antioxidant Nrf2/HO-1 pathway, thereby enhancing endogenous antioxidant defenses and reducing LPS-induced OS in BV2 microglial cells [139]. In human hepatocyte-derived HepG2 cells, tryptanthrin exhibited protective effects against tert-butyl hydroperoxide (tBHP)-induced OS. In this model, tryptanthrin activated the ERK/Nrf2 signaling pathway, thereby enhancing endogenous antioxidant defenses and reducing intracellular ROS levels [140]. Tryptanthrin also showed protective effects against OS in AA + iron-stimulated HepG2 cells. It activated the AMP-activated protein kinase (AMPK)-dependent p38 MAPK signaling pathway. In this model, OS increased ROS levels, which further activated the apoptotic pathway; however, tryptanthrin treatment decreased ROS accumulation and altered key apoptotic proteins by increasing the anti-apoptotic Bcl-2 and decreasing the pro-apoptotic Bax and caspase activation. Similarly, in an animal model of phenylhydrazine (PHZ)-induced acute liver damage, tryptanthrin treatment reduced OS and apoptosis [141].

**Figure 4:**
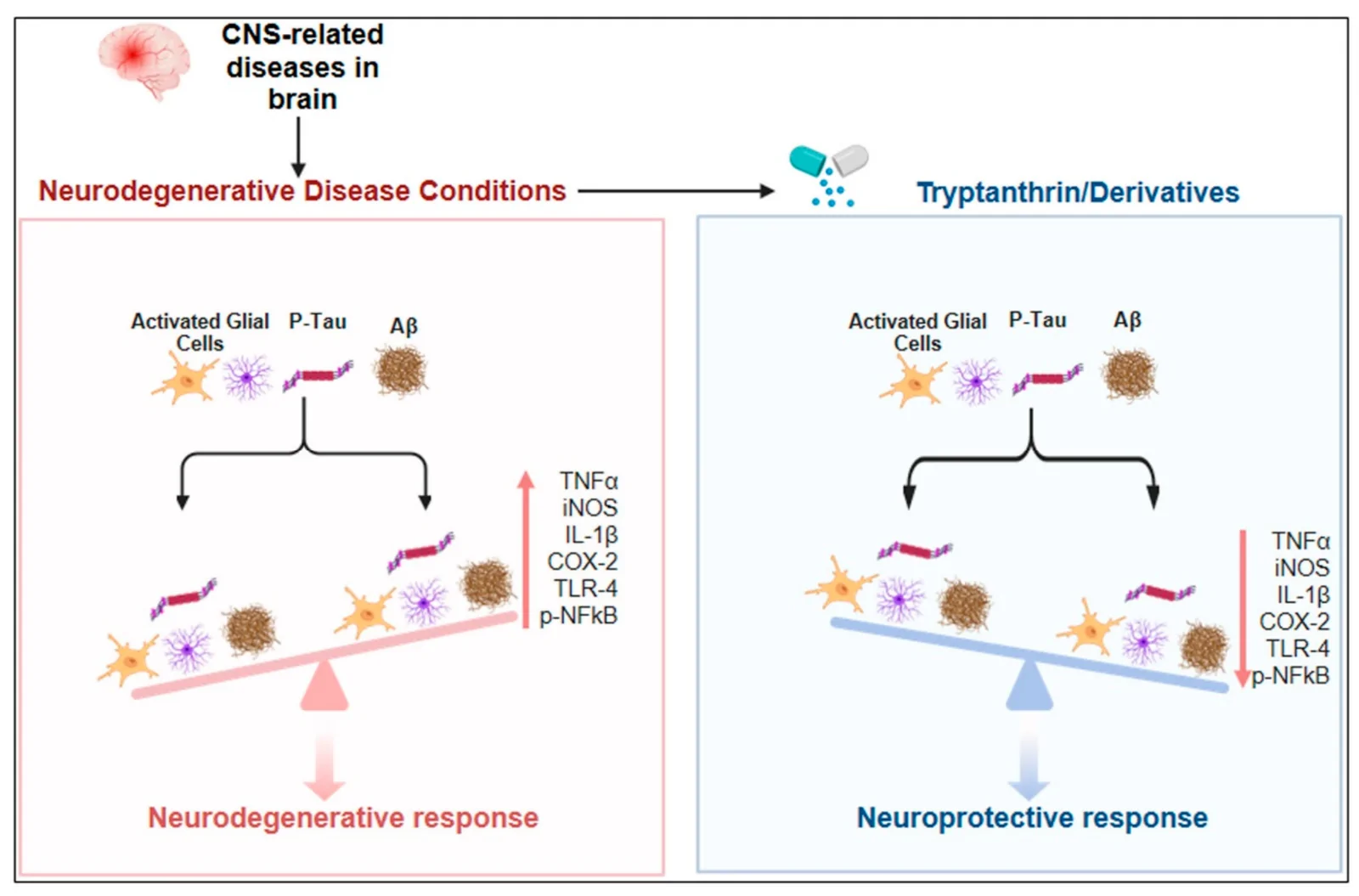
Neuroprotective properties of tryptanthrin and its derivatives in neurodegenerative conditions. Schematic representation of the neuroprotective effects of tryptanthrin and its derivatives in accumulation of pathological proteins, amyloid-β (Aβ), and phosphorylated tau (p-Tau). Pro-inflammatory mediators (TNF-α, IL-1β, IL-6, iNOS, COX-2) are also increased during neurodegeneration. Treatment with tryptanthrin and its derivatives attenuates these pathological changes.

Collectively, the above findings show that tryptanthrin modulates several signaling pathways, including activation of the Nrf2/HO1 pathway and inhibition of inflammation through NF-κB signaling. Furthermore, its ability to reduce tau hyperphosphorylation and Aβ aggregation highlights its potential role against AD. By acting on multiple targets, tryptanthrin may serve as a therapeutic agent for the management of NDs. The molecular mechanisms underlying the neuroprotective effects of tryptanthrin and its derivatives are illustrated in Figure 4.

## 9. Discussion

Neurodegenerative diseases (NDs), including Alzheimer’s disease (AD), Parkinson’s disease (PD), and Huntington’s disease (HD), are the most prevalent neurological disorders. These conditions are strongly associated with aging, and their prevalence continues to rise as the population ages. Currently, NDs have no known cure, and existing therapies mainly relieve symptoms or slow disease progression. Therefore, the development of effective therapeutic approaches that can halt or cure these diseases is urgently needed.

Natural compounds and their derivatives have attracted significant research interest for the development of innovative therapies due to their various biological activities. Natural compounds have the ability to target several pathogenic pathways involved in neurodegeneration. Alkaloids are naturally derived and pharmacologically active compounds and have revealed protective properties in several diseases. Alkaloids show potential for neuroprotection by regulating several pathways, including oxidative stress, neuroinflammation, protein aggregation, mitochondrial dysfunction, and neuronal cell death. This review summarizes that numerous alkaloids have multitarget beneficial neuroprotective effects in experimental models of several neurodegenerative conditions, including AD, PD, HD, and traumatic brain injuries (TBI).

Furthermore, this review highlights the neuroprotective potential of tryptanthrin and its derivatives in several neurodegenerative diseases. Preclinical evidence indicates that tryptanthrin and its derivatives can modulate multiple signaling pathways involved in neuronal injury and neurodegeneration, thereby exerting antioxidant, anti-inflammatory, and anti-apoptotic effects. Overall, the current preclinical evidence shows that tryptanthrin and its derivatives are strong candidates for future exploration in neurodegenerative disorders. However, translating these findings into therapeutic applications remains a major current challenge, and future perspectives are discussed in the following section.

## 10. Current Challenges and Future Perspectives

Despite the remarkable protective properties of tryptanthrin and its derivatives in neurodegenerative diseases, various difficulties remain before their possible therapeutic usage in clinical settings. The current available data are taken from in vitro and animal research investigations, which revealed differences in models, dosages, treatment duration, and route of administration.

Another major challenge is the pharmacokinetic studies of tryptanthrin and its derivatives. Currently, there is insufficient information on the blood–brain barrier (BBB) permeability, optimum therapeutic dose, oral bioavailability, metabolic stability, and long-term safety. These are important and necessary parameters for developing a novel drug that targets the central nervous system.

Future research on tryptanthrin and its derivatives should include safety, toxicity studies, pharmacokinetic studies, optimization of dose, route of administration, and validation of the neuroprotective effects in clinically relevant disease models. In addition, further research is required to identify the molecular targets and signaling pathways through which tryptanthrin and its derivatives exert neuroprotective effects in the brain. A better understanding of their mechanisms of action will help to clarify how these compounds modulate key signaling pathways involved in the development and progression of neurodegeneration. Finally, well-designed clinical trials will be required to prove their safety, effective dose, and therapeutic effectiveness in humans.

## 11. Conclusions

This review emphasizes the accumulating evidence that tryptanthrin is a potential alkaloid for the treatment of neurodegenerative disorders. Although current research suggests that it is a strong candidate for the treatment of neurodegenerative diseases in pre-clinical studies, there are also limited studies about pharmacokinetics and long-term safety.

Future studies will be required to discover the molecular mechanisms of tryptanthrin against neurodegenerative diseases. These initiatives may help with the development of tryptanthrin and its derivatives as potential treatments for neurodegenerative diseases.

## Figures and Tables

**Table 1:** Tryptanthrin derivatives.

Tryptanthrin Derivatives	References
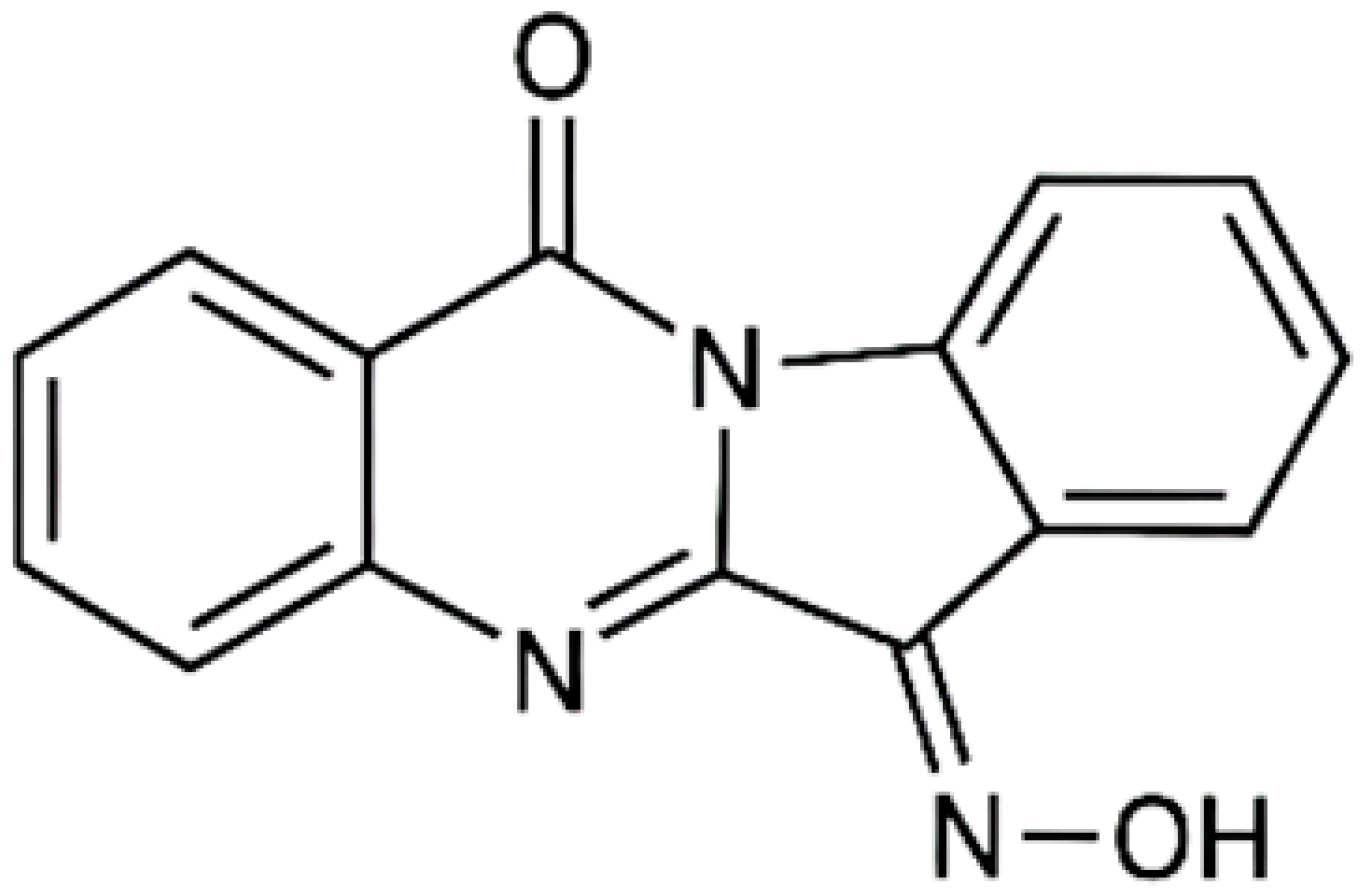 Tryptanthrin-6-oxime	[110]
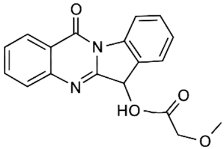 Phaitanthrin-B	[111]
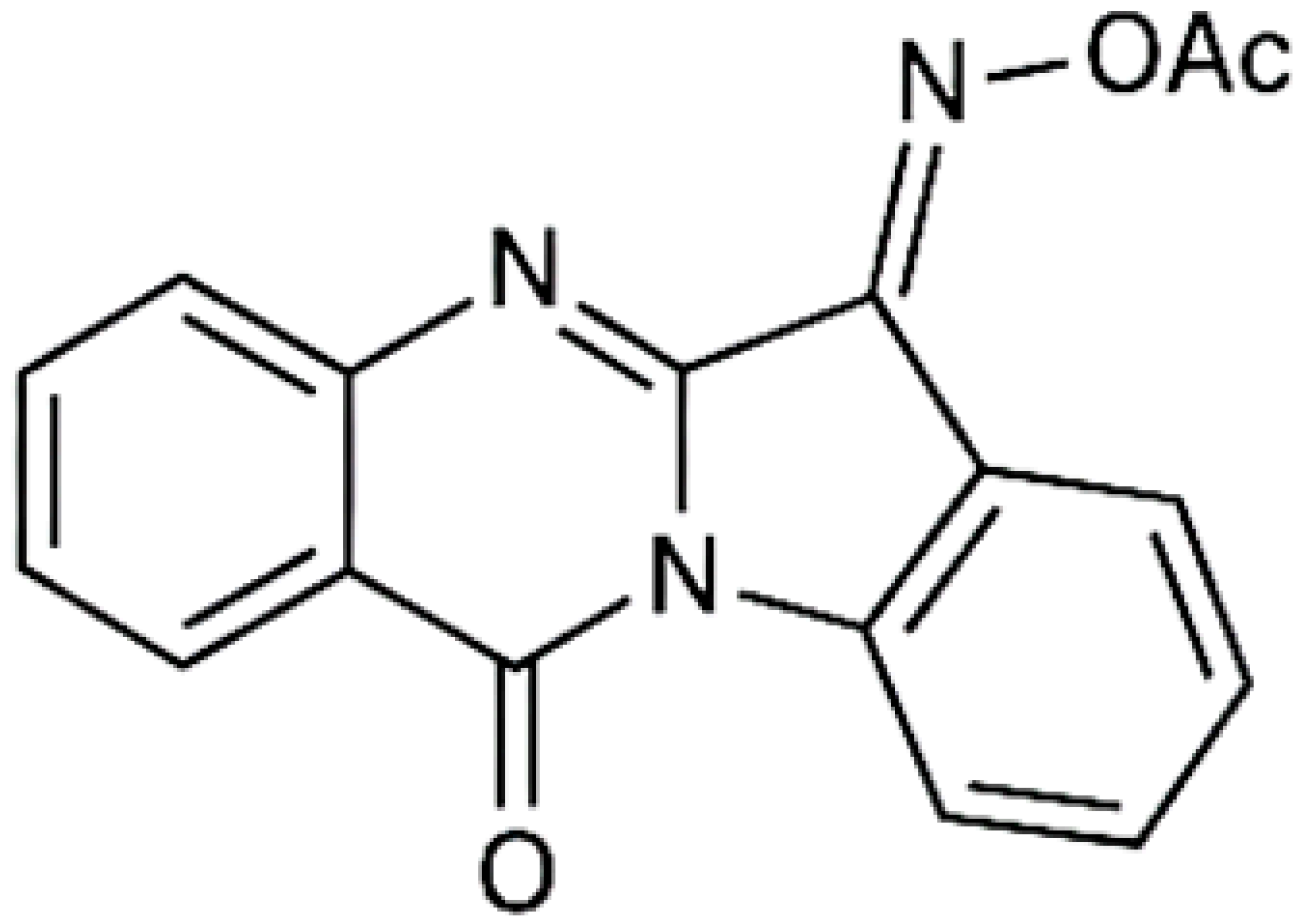 Acetyl-substitute-6-oxime	[112]
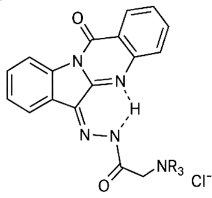 Mostotrin	[113]
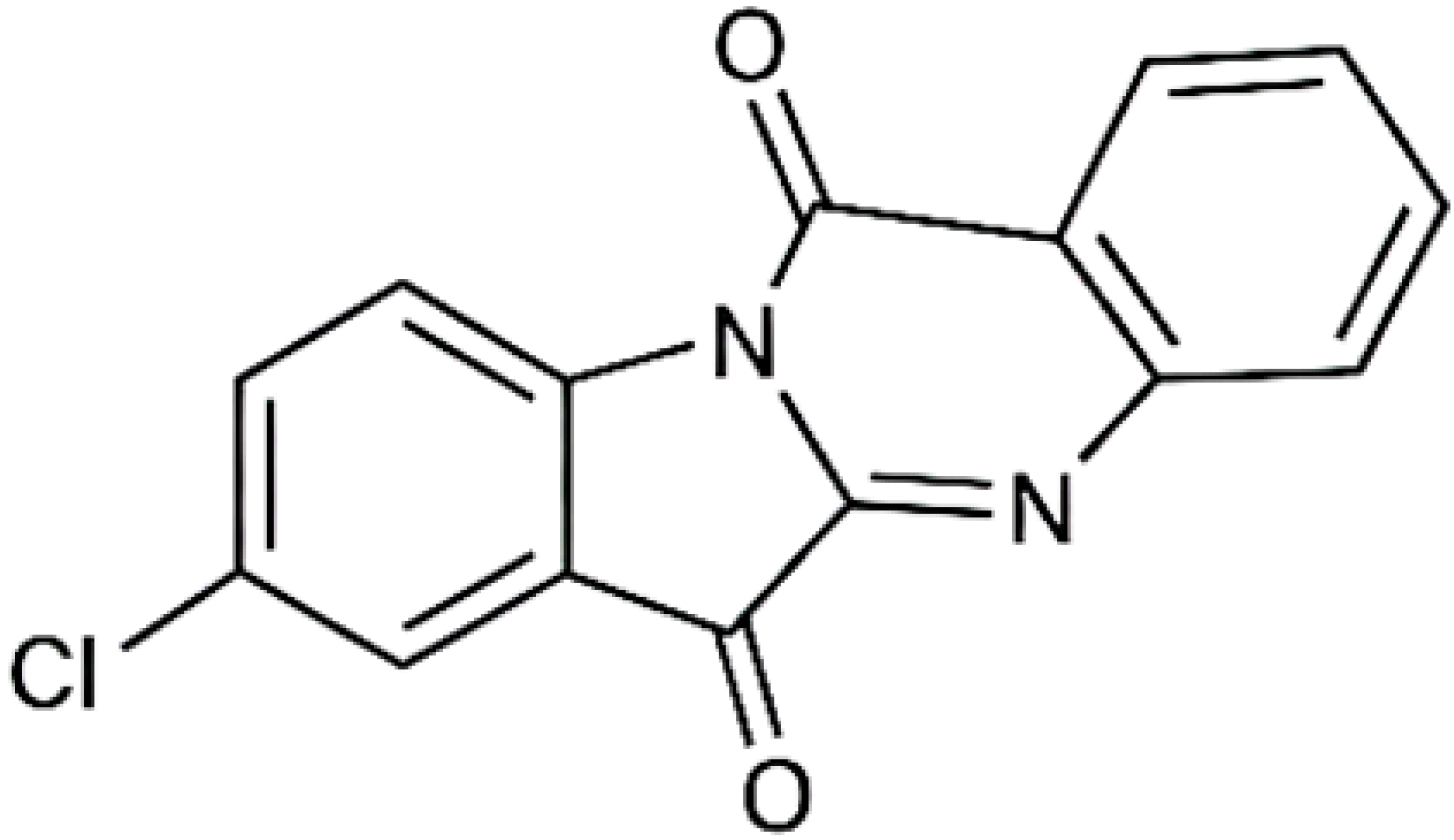 Chlorotrytanthrin	[114]
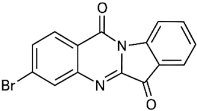 Bromo-Tryptanthrin	[115]
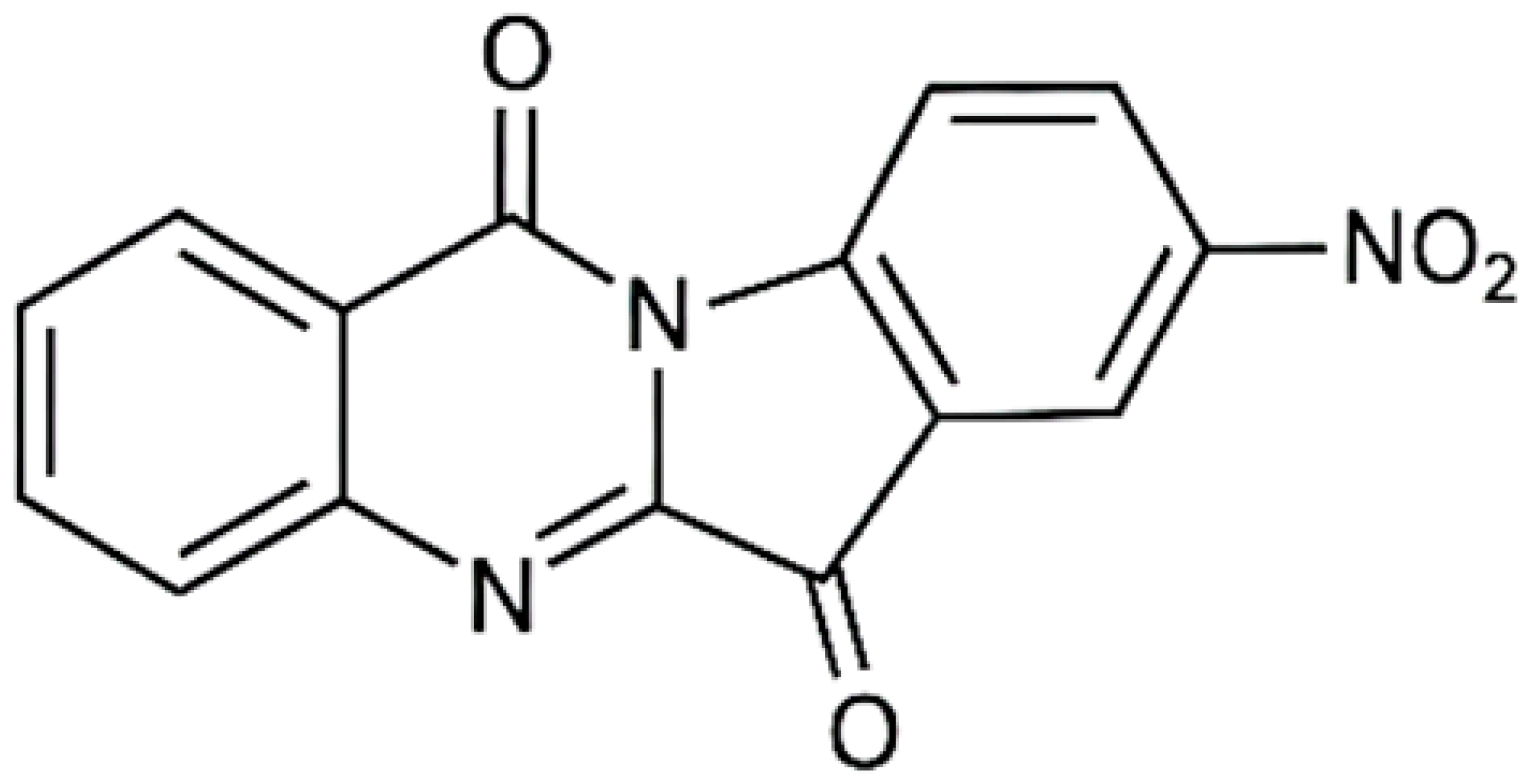 Nitrotryptanthrin	[116]
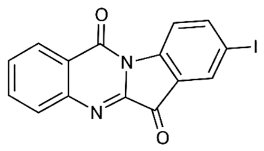 Iodotryptanthrin	[117]
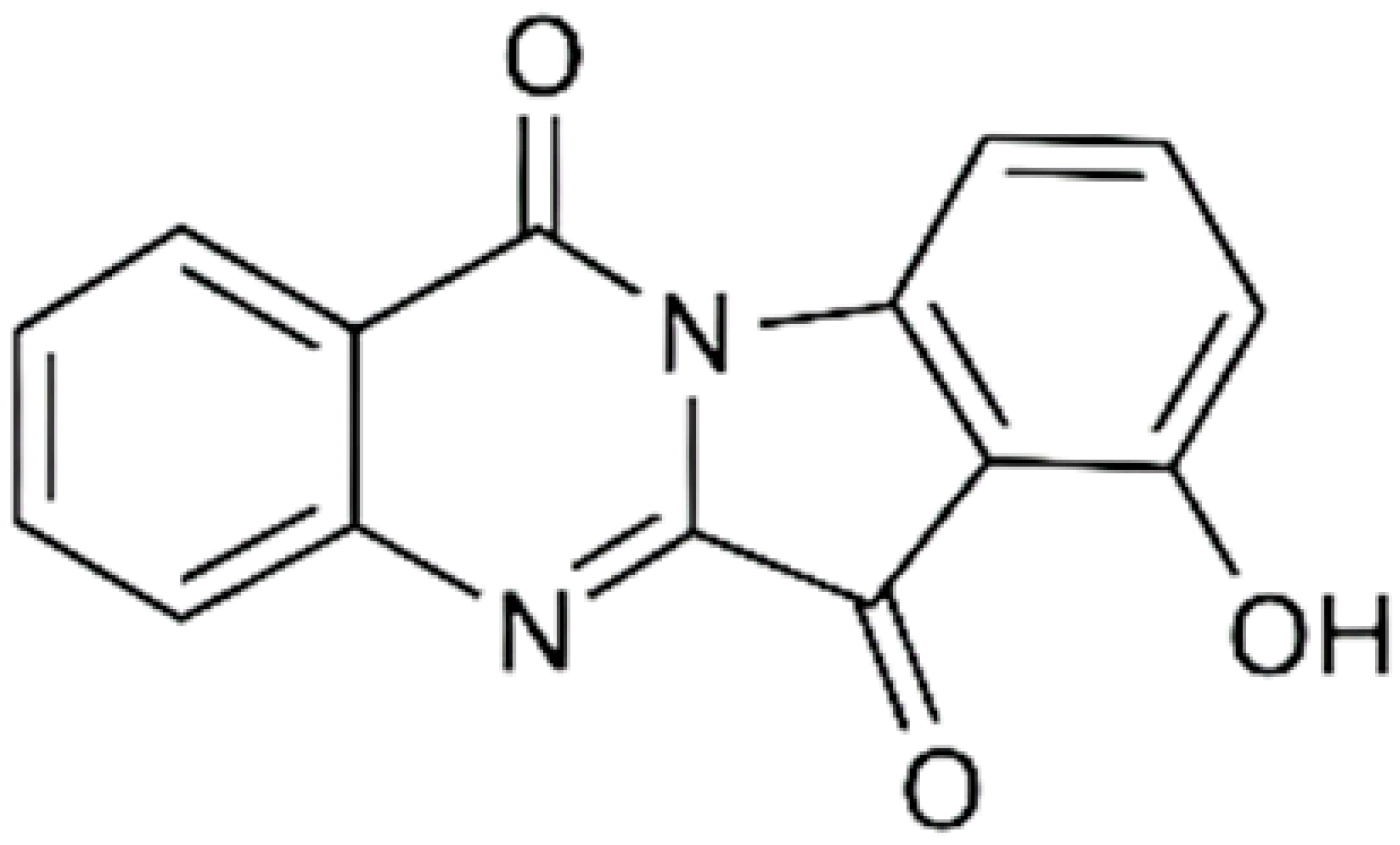 Phaitanthrin-C	[38]
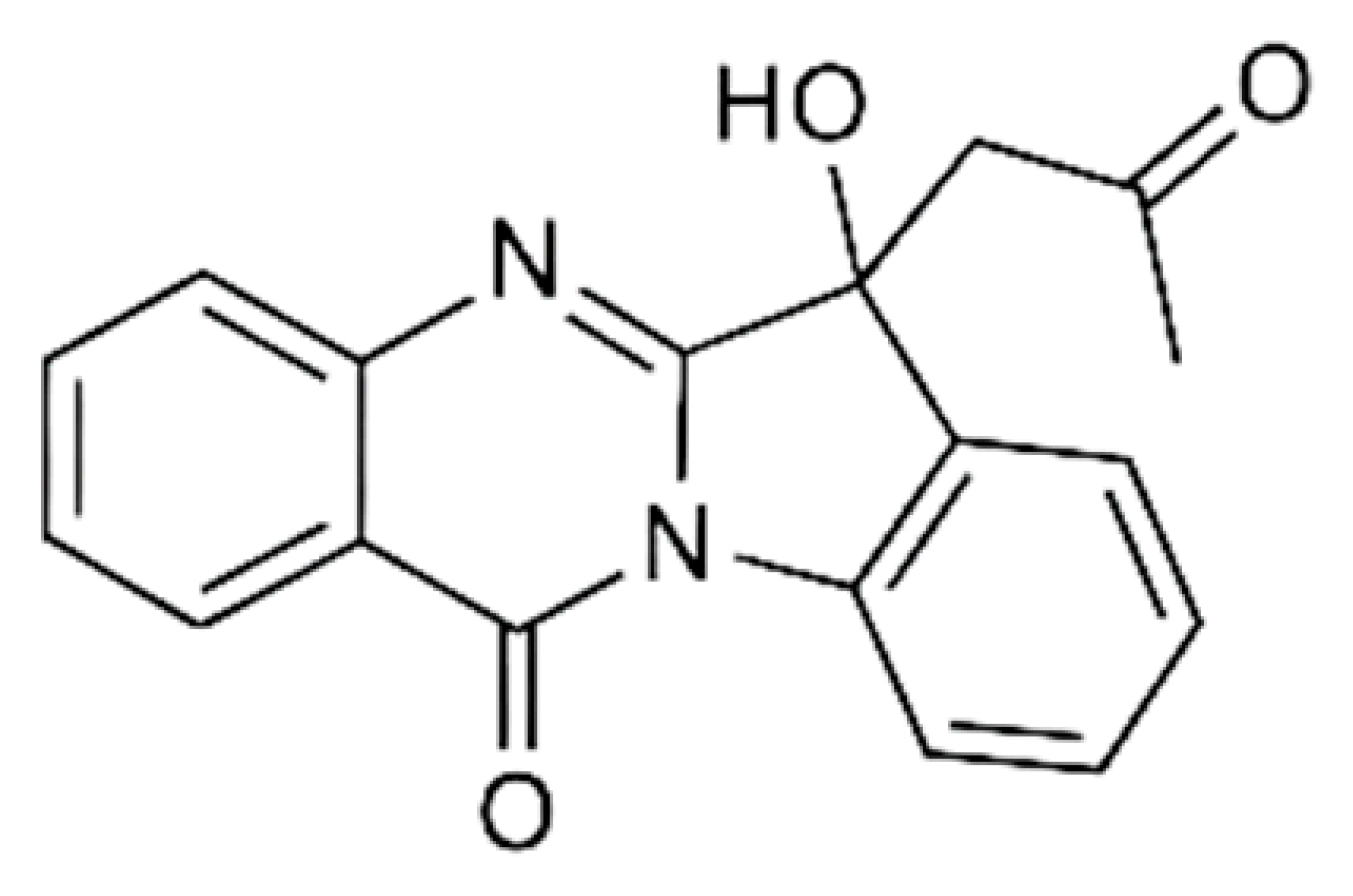 Phaitanthrin-A	[118]

## Data Availability

No new data were created or analyzed in this study. Data sharing is not applicable to this article.

## Abbreviations

The following abbreviations are used in this manuscript:

AD	Alzheimer’s disease
PD	Parkinson’s disease
HD	Huntington’s disease
NDs	Neurodegenerative diseases
CNS	Central nervous system
ROS	Reactive oxygen species
NRF2	Nuclear factor erythroid 2–related factor 2
HO-1	Heme oxygenase-1
p38 MAPK-p38	mitogen-activated protein kinase
COX-2	Cyclooxygenase-2
iNOS	Inducible nitric oxide synthase
IL-1β	Interleukin-1 beta
TNF-α	Tumor necrosis factor-alpha
